# A review of documents prepared by international organizations about influenza pandemics, including the 2009 pandemic: a bibliometric analysis

**DOI:** 10.1186/s12879-018-3286-3

**Published:** 2018-08-08

**Authors:** Feng Liang, Peng Guan, Wei Wu, Jing Liu, Ning Zhang, Bao-Sen Zhou, De-Sheng Huang

**Affiliations:** 1 Department of Epidemiology, School of Public Health, China Medical University, Shenyang, 110122 China; 2 Department of Mathematics, School of Fundamental Sciences, China Medical University, Shenyang, 110122 China

**Keywords:** Pandemic influenza, H1N1, Sectors, Intervention, International organization

## Abstract

**Background:**

World Health Organization (WHO), the World Bank, UN System Influenza Coordination (UNSIC) and other international organizations released a series of documents to fight against the influenza pandemic. Those documents have great significance on guiding influenza pandemic preparedness and responses and providing a multilevel, multi-directional influenza pandemic prevention and control network for their member countries. This study focuses on the above-mentioned influenza pandemic preparedness guidelines with the aim of exploring the roles of the society, defining the relationship of different interventions and evaluating the planning on influenza pandemic preparedness.

**Methods:**

Documents about pandemic influenza preparedness were retrieved from the official websites of the following three international organizations, World Health Organization (WHO), the World Bank, UN System Influenza Coordination (UNSIC) with the key words ‘pandemic’, ‘influenza’ and the Boolean combinations of these words as the retrieval strategy. Guidelines, research study and meeting reports were included in the study. The categories of the ministries/departments involved and their roles/responsibilities in pandemic influenza preparedness were summarized. Word frequency of selected vocabularies about pandemic influenza preventive measures were collected from the documents and the correlations between the word frequency of these measures were analyzed. Ochiai coefficient was employed to show the correlation between the word vocabularies.

**Results:**

A total of 38 records on the topic of pandemic influenza preparedness were included. The responsibilities of the whole-of-society mentioned in the international organizations’ documents varied across the 2009 influenza pandemic period. Meanwhile, it had been emphasized that a comprehensive influenza prevention and control plan in every sector should be developed and evaluated. Because various measures were emphasized in the guidelines after 2009 pandemic influenza, the correlations between the word frequencies of the various influenza preventive measures became stronger after the pandemic influenza.

**Conclusions:**

Responsibilities of ministries of education, ministries of energy, ministries of agriculture and animal health, ministries of communication and the business sector in the pandemic influenza preparedness were described more comprehensively in the international organizations’ documents in 2017. Better understanding the variations of the guidelines delivered by international organizations would be useful for the member countries to strengthen their influenza control network.

**Electronic supplementary material:**

The online version of this article (10.1186/s12879-018-3286-3) contains supplementary material, which is available to authorized users.

## Background

As the unpredictable but recurrent events, influenza pandemics have rapid also severe consequences on human health and the economy in all parts of the world [1]. The influenza A(H1N1) 2009 pandemic was the first pandemic influenza in the 21st century, the outbreak of the new A(H1N1)pdm09 virus triggering worldwide attention to influenza [2]. During the 2009 pandemic, over 600,000 confirmed cases were reported having been infected A(H1N1)pdm09 virus worldwide [3], more than 208 countries and territories have been affected [4].

The advance planning and preparedness play important roles in mitigating the impact of an influenza pandemic [5–7]. Previous reports have indicated that the significant improvements in influenza pandemic preparedness between 2008 and 2012 in Central America [8] and the preparedness planning might strengthen the collective, multilevel responses to future public health crises [9]. The investment in pandemic influenza preparation has been proved to contribute the improvement of capacity ability in health systems in Asia [10].

World Health Organization (WHO), the World Bank, UN System Influenza Coordination (UNSIC) and other international organizations released a series of documents to fight against the influenza pandemic. Those documents have great significance on guiding influenza pandemic preparedness and responses and providing a multilevel, multi-directional pandemic prevention and control network for their member countries.

International organizations have been central to handling different aspects of pandemic influenza preparedness, from macro-level policy design to micro-level specific preventive measures. However, despite their importance, we lack a systematic understanding of changes of the guidelines or documents regarding pandemic influenza preparedness across the 2009 pandemic. In this context, this article seeks to clarify the roles and responsibilities of each sector involving in pandemic influenza preparedness via the content analysis of the guidelines, the changes of the guidelines across the 2009 pandemic and the theme of assessment that mentioned in the documents. Based on our findings, it is promising for the member countries of the international organizations to establish the framework to take stock of influenza pandemic readiness and address the coordination challenges.

## Methods

### Inclusion and exclusion criteria

The present review focused on the documents released by the international governmental organizations within the UN system about pandemic influenza preparedness. The inclusion criteria of the international organizations were as follows, a) the international organization was within the UN system; b) the international organization provided leadership, financial assistance, cooperation and coordination for pandemic influenza preparedness, c) the international organization dealt with global challenges of influenza control in the whole population. The exclusion criteria of the international organizations were a) the regional office of the international organization, b) main attention on animal health, such as World Organization for Animal Health, and c) main attention on part of the whole population, such as UNICEF.

On the basis of the above inclusion criteria and restrictions, World Health Organization (WHO), The World Bank, UN System Influenza Coordination (UNSIC) Office were included in the study. WHO is the directing and coordinating authority on international health within the United Nations’ system; the mission of the World Bank is to end extreme poverty and promote shared prosperity; the primary purpose of UNSIC Office is to ensure cooperation and coordination within the UN system in support of different initiatives underway to address the avian flu epidemic and the threat of a human pandemic.

Documents about pandemic influenza preparedness were retrieved from the official websites of these three international organizations with the key words ‘pandemic’, ‘influenza’ and the Boolean combinations of these words as the retrieval strategy. Guidelines, research study and meeting reports were included in the study. The last search date was July 3, 2017. The full text of each document was downloaded and screened according to the information obtained from the title, abstract or main text. Only the documents reporting pandemic influenza preparedness were included. News and comments were excluded from the analysis.

### Theme summary and correlation analysis

After reading the full text, the basic information of the included documents was described, the categories of the ministries/departments involved and their roles/responsibilities in pandemic influenza preparedness were summarized. In the process of vocabularies extraction, the initial vocabularies were determined by consulting experts, and the final vocabularies that were submitted to the word frequency analysis were determined due to their existence in equal to and more than half of the documents. To accurately extract and count the bibliographic information from worldwide international websites, the co-occurrence matrix was generated and basic data were submitted to subsequent statistical analysis. To ensure the reliability, two authors (FL and PG) independently screened each document and the overlapped words were decided first and the differences were settled after discussion, the information extraction process was checked by a third reviewer (DSH).

### Statistical analysis

Word frequency of selected vocabularies about pandemic influenza preventive measures were collected from the documents and the correlations between the word frequency of these measures were analyzed. In order to reduce the influence of the maximum or minimum of frequency between the vocabularies on the correlation, Ochiai coefficient was employed to show the correlation between the two vocabularies with the calculation formula Ochiai coefficient = $\frac{AB}{\sqrt{A^{\ast }B}}$, where AB is the frequency of co-occurrence between words A and B; A is the frequency of occurrence of the word A; B is the frequency of occurrence of the word B. The calculation was done by using the software Statistical Product and Service Solutions (SPSS 12.0 for windows, SPSS Inc., Chicago, IL, USA).

## Results

### Documents search and the characteristics of the included documents

**Fig. 1:**
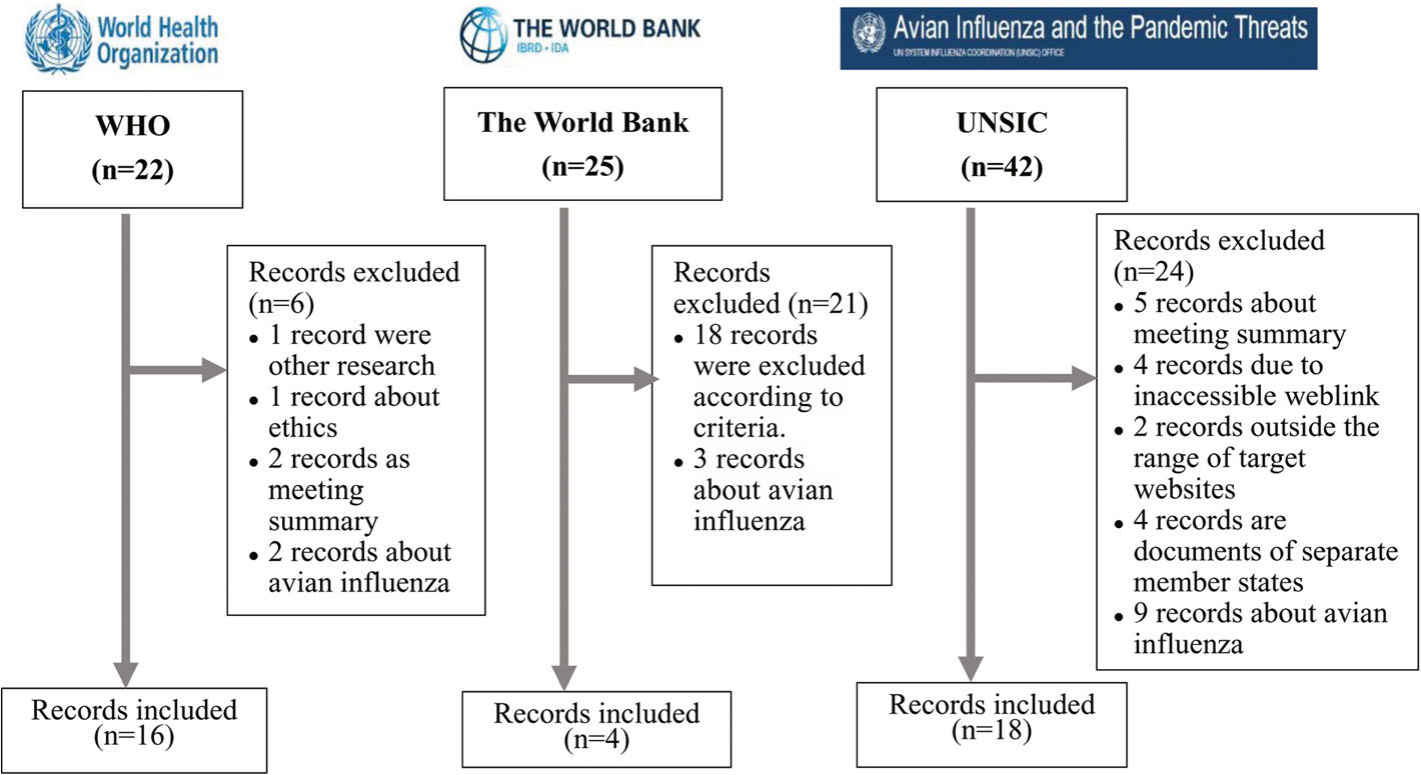
Flow chart of document search and selection process about pandemic influenza preparedness

Based on our search strategy, a total of 89 records on the topic of pandemic influenza preparedness were first identified from the official websites of these three international organizations. The major paths to retrieve the included documents from the three international organizations’ official websites were as follows: WHO (http://www.who.int/en/) > Health topic >Influenza >Public health preparedness > List of documents on Pandemic; UNSIC (http://www.un-influenza.org/) > topic > Pandemic Preparedness Guidance for the UN System; UNSIC > Resources > key documents; The World Bank > What We Do > Development Knowledge > Research & Publication. There were 38 records included in the bibliometric analysis, besides WHO, UNSIC and the World Bank, all the involved organizations included FAO, ICAO, ILO, IOM, OCHA, OIE, UNDP, UNFPA, UNHCR, UNICEF, UNWTO, WFP, ADPC, K. I. Asia and GTZ. The flowchart of the literature search and selection process according the inclusion criteria is shown in Fig. 1.

The basic information of the included 38 documents are described from six aspects: source, author, publishing date, title, theme, number of pages in its original format. The earliest document was released in 2004, and the latest document was released in 2017. There were 13 documents before the influenza pandemic (before April 2009), 6 documents during the pandemic period (April 2009 to August 2010), 17 documents after the pandemic (after August 2010), and the publishing dates of two documents could not be identified. The length of the documents in this field is also a good indicator of international efforts to improve influenza pandemic preparedness. The length of each document was judged by the number of pages which ranged from 1 to 180 and the median of pages was 44.5. The characteristics of the included documents are presented in Additional file 1: Table S1.

### Whole-of-society pandemic readiness

WHO first proposed whole-of-society pandemic readiness in WHO guidelines for pandemic influenza preparedness and response in 2009 [7]. Detailed information on the whole-of-society approach were provided in the documents. After consulting a range of experts their opinions of pandemic influenza and discussion, WHO developed the recommendations on pandemic influenza preparedness, which was not evidence-based [11]. WHO released Pandemic Influenza Risk Management-WHO Interim Guidance in 2013 and Pandemic Influenza Risk Management in 2017, which took account of lessons and experience learnt from the influenza A(H1N1) 2009 pandemic and of other developments in relevant fields [5, 6]. The roles and responsibility of whole-of-society across the influenza pandemic period are shown in Table 1, where the differences of the documents’ context between 2009 and 2017, are described. The roles and responsibilities in 2017 are described more comprehensively for the following sectors: ministries of education, ministries of energy, ministries of agriculture and animal health, ministries of communication and the business sectors. Meanwhile, it has been emphasized that a comprehensive influenza prevention and control plan in every sector should be developed and evaluated.

**Table 1:** The roles and responsibilities of whole-of-society proposed by WHO in 2009 and the addition in 2013 and 2017

	Sectors	Roles and responsibility in 2009^a^	Added roles and responsibility in 2013^b^ and 2017^c^
Government	Central government	1. Leader; 2. Enact or modify legislation and policies; 3. Prioritize and guide the allocation and targeting of resources; 4. Provide planning information and framework; 5. Define, oversee, and coordinate preparedness actions.	1. Conduct training to ensure effective dissemination at all levels; 2. Design and implement exercises to test plans and encourage community mobilization
	Ministries of Transportation	1. Minimize infection risks and staff absences in vital transportation plots	1. Mechanisms for communication and education of public transportation users
	Ministries of Finance	1. Maintain essential financial services; 2. Conduct testing of systemic resilience to pandemic risk	1. National-level financial planning for pandemic risk management; 2. The mechanisms to draw down emergency funding for interventions should be tested prior to a pandemic.
	Ministries of Justice	1. Consider what legal processes could be suspended and make alternative plans to operate courts during pandemic.; 2. Minimize the spread of infection in prisons and other institutions under their authority.	1. Maintain all essential legal and administrative operations during a pandemic; 2. Plans for infection control and risk reduction in facilities should be tested in conjunction with the Ministry of Health plans.
	Ministries of Defense	1. Release and mobilize military assets	1. Based on Ministry of Health planning assumptions and risk assessment
	Ministries of Education		1. Surveillance of absenteeism in schools; 2. Linking of school surveillance systems with the Ministry of Health.
	Ministries of Energy		1. Well-developed and well-exercised preparedness plans; 2. Alternative plans for energy supplies.
	Ministries of Communication		1. Ensure communication channels open at the time of crises; 2. Develop a national communication plan
	Ministries of Agriculture and Animal Health		1. Surveillance and monitoring of non-seasonal influenza viruses; 2. Decrease the exposure of humans to influenza viruses at the human–animal ecosystem interface.
	Ministries of Health	1. Provide reliable information on the risk, severity, and progression of a pandemic and the effectiveness of interventions used; 2. Provide health care; 3. Reduce the spread of influenza; 4. Protect and support health-care workers.	
Business Sectors			1. Medical supplies and services; 2. Monitor absenteeism; 3. Reduce population density in shopping areas; 4. Protect employees; 5. Provide accurate and timely communication messages; 6. Provide personal protective equipment and training
Civil society and community-based organizations		1. Translate scientific and government messages and recommendations; 2. Build public confidence, disseminate information, and identify people at risk; 3. Provide community-based services	1. Maintain or expand their essential services
Individuals and families		1. For families, have access to reliable information from sources; 2. For individuals, assist the others; 3. Adoption of individual and household measures, such as covering coughs and sneezes, hand washing, and the voluntary isolation of persons with respiratory illness	

^a^ World Health Organization. Pandemic Influenza Preparedness and Response, 2009

^b^ World Health Organization. Pandemic Influenza Risk Management, 2013

^c^ World Health Organization. Pandemic Influenza Risk Management, 2017

Whole-of-society pandemic readiness are also emphasized in the World Bank and UNSIC. WHO elaborated on it while the World Bank and UNSIC did not. The World Bank focused on the source, allocation and disbursement of funding for the pandemic influenza preparedness, and shared some useful lessons on “good health at low cost” in Sri Lanka [12]. UNSIC paid attention to all aspects of flu such as funding, risk assessment, medical guidance, country coordination, urgent support, simulation exercise and etc.

### Words frequency analysis of the intervention on pandemic influenza

After summarizing the vocabularies on prevention and control measures across the influenza pandemic period in the included documents, the frequencies of selected vocabularies were analyzed and sent to the correlation analysis. Two documents were excluded in the word frequency analysis due to the unknown publication date. After first round of vocabulary screening, 6 words were excluded due to their absence in more than half of the 36 included documents to get a more accurate result. The final included vocabularies and the co-occurrence of word frequency in the three phases are indicated in Table 2. The Ochiai coefficient varies across the pandemics influenza period, 8, 20 and 16 pairs of vocabularies exceed 0.5 in the period that before, during and after pandemic, respectively. Prior to 2009, the focus on influenza that with pandemic potential was mainly on the avian influenza subtype A(H5N1). Biosecurity was the most important activity before 2009 and the correlations between the word frequencies of the various preventive measures were not strong. During the pandemic (2009–2010), a ‘whole-of-society’ approach to pandemic influenza preparedness was proposed by WHO in 2009, which resulting in more frequent cooccurrence of the measures in the documents. The following four words’ frequencies were closely related to each other: plan, policy, communication and surveillance.

**Table 2:** Ochiai coefficients similarity matrix of high frequency keywords (before / during / after influenza pandemic) about pandemic Influenza Intervention

	Plan	Policy	Communication	Surveillance	Risk assessment	Investigation	Education	Hygiene	Quarantine	Vaccine/ vaccination	Antiviral	Essential service
Plan	1											
Policy	0.23/0.44/0.33	1										
Communication	0.53/0.71/0.58	0.29/0.61/0.52	1									
Surveillance	0.51/0.67/0.52	0.36/0.60/0.53	0.62/0.75/0.71	1								
Risk assessment	0.13/0.13/0.32	0.22/0.31/0.44	0.24/0.19/0.34	0.26/0.20/0.34	1							
Investigation	0.26/0.17/0.21	0.33/0.39/0.54	0.42/0.24/0.32	0.49/0.26/0.37	0.51/0.71/0.27	1						
Education	0.19/0.31/0.24	0.32/0.51/0.54	0.36/0.43/0.34	0.38/0.42/0.40	0.49/0.44/0.29	0.43/0.56/0.72	1					
Hygiene	0.14/0.26/0.21	0.23/0.32/0.40	0.25/0.36/0.31	0.26/0.37/0.27	0.67/0.47/0.22	0.36/0.67/0.42	0.57/0.67/0.57	1				
Quarantine	0.22/0.14/0.16	0.45/0.32/0.38	0.32/0.20/0.26	0.33/0.19/0.28	0.27/0.76/0.25	0.28/0.53/0.62	0.48/0.38/0.67	0.34/0.40/0.41	1			
Vaccine/vaccination	0.29/0.67/0.37	0.43/0.49/0.40	0.21/0.77/0.47	0.37/0.58/0.50	0.13/0.17/0.50	0.25/0.21/0.29	0.15/0.37/0.35	0.13/0.31/0.25	0.25/0.16/0.27	1		
Antiviral	0.31/0.35/0.31	0.43/0.30/0.43	0.31/0.49/0.47	0.52/0.46/0.51	0.22/0.39/0.40	0.39/0.34/0.39	0.28/0.46/0.33	0.20/0.60/0.30	0.47/0.37/0.28	0.55/0.43/0.54	1	
Essential service	0.24/0.26/0.18	0.25/0.43/0.46	0.30/0.28/0.31	0.44/0.22/0.33	0.33/0.51/0.43	0.45/0.53/0.64	0.46/0.45/0.58	0.32/0.51/0.39	0.40/0.54/0.49	0.22/0.18/0.32	0.38/0.38/0.41	1

### Theme of assessment via content analysis

Assessment of the prevention and control plan for the pandemic influenza has been mentioned in 10 of the 38 included documents. Among the ten documents, the content is consistent in many aspects, with some differences in the level of detail for the description. Eight documents were released by WHO, focusing on the ‘whole-of-society’ pandemic readiness; the rest two documents were issued by the World Bank and UNSIC, focusing on the pandemic risk, continued vigilance and investments. Five categories of assessment theme were summarized as follows: assessment of pandemic severity, risk assessment, assessment of national planning, simulation exercises on influenza pandemic responses and effectiveness of public health measures. A summary of the essential content in each category is provided in Table 3.

**Table 3:** Assessment theme and the content for the prevention and control of the pandemic influenza

Assessment Theme	Details
Assessment of pandemic severity	1. Transmissibility: virological factors and epidemiological observations; 2. Seriousness of disease; 3. Impact: socioeconomic impact [5].
Risk assessment	1. Hazard assessment: identifying, reviewing and ranking influenza viruses of concern; 2. Exposure assessment: epidemiological and susceptibility factors; 3. Context assessment: social, technological and scientific, economic, ethical, and policy and political factors; 4. Risk characterization [5].
Assessment of national planning	1. Planning and coordination; 2. Situation monitoring and assessment; 3. Prevention and containment; 4. Health system response; 5. Communication [27].
Simulation exercises on influenza pandemic responses	1. Orientation: an informal discussion designed to familiarize participants with plans, roles and procedures with a focus on questions of coordination and assignment of responsibilities; 2. Drill: developing and maintaining skills in a single response procedure; 3. Table-top exercise (TTX): a discussion held around a conference or round table over the space of 2 or 3 h; 4. Functional exercise: creating a situation and facilitating a ‘real’ response, and may include such activities as activating command centres, documenting actions and decisions, completing real forms, issuing real communications and responding to simulated media or other questions; 5. Full-scale exercise: focusing on the operational capability of emergency response and management systems [29].
Effectiveness of public health measures	1. Economic effects; 2. Influenza-like illness(ILI), hospitalization rates or laboratory-confirmed cases, incidence rate ratios; 3. Change of behavior [30].

## Discussion

The comprehensive, coordinated, whole-of-society approach to pandemic preparedness is necessary and crucial to the success of influenza control program [5]. The communities of international organizations have paid increasing attention to bringing member countries, industry and some other stakeholders together to implement the global approach to pandemic influenza preparedness. In this context, the present bibliometric analysis may serve as a starting point for further improvement of international guidance regarding pandemic influenza preparedness. We focused on the official documents of international organizations related to the topic of pandemic influenza control from the quantitative, qualitative and content’s points of view and clearly shows the variations of these documents.

The consequences of the pandemic influenza for society and economy will not be more serious, if the governments, businesses, civil society, individuals and families have planned preparedness to how they can maintain their essential services and communicate disease information validly. It has been emphasized by the ‘whole-of-society’ approach to pandemic influenza preparedness that all sectors of society played significant roles [7]. The differences of the sector responsibilities and measures description that mentioned in the existing guidelines across the pandemic period provided some hints of guidelines modification. Compared to 2009, the roles and responsibilities in 2017 were described more comprehensively in ministries of education, ministries of energy, ministries of agriculture and animal health, ministries of communication and the business sector. Lessons learned from the 2009 H1N1 pandemic influenza action, the roles and responsibilities of the different departments were refined more clearly.

As shown in Table 1, the training items were added for the central government and business sectors in the version of 2013 and 2017. The nation-level financial planning and emergency funding mechanisms for pandemic influenza was added for ministries of finance in the version of 2013 and 2017. Because of the urgency and complexity of the pandemic H1N1 situation, a flexible financing framework embracing a variety of funding mechanisms was proposed as the best means of providing financial support to least resourced countries [13, 14]. Participating countries were seeking support to enable them to strengthen their readiness and response to H1N1 pandemic influenza. Capacity development is central to ensure that the workforce is well equipped to implement influenza response planning. Thus, funding and training are crucial to a national pandemic preparedness plan [5].

It has been proved in the global polio eradication action that the effectiveness of technology depends on national political commitment, security assurance, social support, religious support, local public support, project management and community participation [15, 16]. Influenza pandemic preparedness strategies and polio eradication plan share many common aspects, including extensive surveillance and laboratory networks, preparedness and response to epidemics, extensive communication, social mobilization networks and etc. As expected, the results of the present study provide some hints or experience of the prevention and control of pandemic influenza.

There have been some studies focusing on the intervention of pandemic influenza, such as communication, vaccination coverage, gas mask distribution, public health surveillance, knowledge and practices of intervention [17–26], while the correlations among these interventions were not discussed sufficiently. As the influenza control measures were different across pandemic influenza and the internal links between the measures are vague, the research adopted a word frequency analysis to explore the linkage of various influenza control measures. Information communication and disease surveillance provide essential disease information for better planning and decision making, which has been clearly indicated by the Ochiai coefficients. The higher correlations between the word frequencies after the pandemic influenza suggested that the relationships among the various measures were emphasized.

Assessment is an important part of pandemic influenza preparedness, which cannot be ignored. Early and comprehensive information on the severity of an influenza pandemic and risk assessment can help support decision-making at global and country levels. The five aspects of assessment of national planning have been identified as planning and coordination, situation monitoring and assessment, prevention and containment, health system response and communication [27]. A good planning requires simulation exercises to ensuring the feasibility of the plan [28]. WHO has provided 5 kinds of simulation exercises, each of them having strengths and weakness. Of the five types of exercises, an orientation is the simplest and costs the least, while the table-top exercise (TTX) is the workhorse, ranging in scope and complexity. The full-scale exercise is the most comprehensive also the most cost one [29]. Decision makers can choose the most suitable one according to their situation. Referring to effectiveness of public health measures, it is also difficult to evaluate the effectiveness of individual measures because it was difficult to determine what to measure and it often combined with other interventions [30].

There are several limitations in the present study which may encourage further research efforts. Firstly, although the correlations between the word frequencies of influenza control-related vocabularies in the included documents were analyzed, the interaction of different preventive measures could not be identified. Better understanding the role of each preventive measures in the influenza control network will be considered in our future work. Secondly, the included influenza control-related measures were summarized from the documents released by international organizations, however, the implementation of these measures in the real world is unknown. There will be no response if funds are not set aside for rapid and proper action, moreover, personnel who are properly trained are needed. The governments should plan this and set aside special pandemic influenza preparedness budget when they prepare their yearly budget. Cost-benefit analysis may help to provide some evidence in the future. Thirdly, different experts were invited by each international organization according to its own interest to provide their opinions about pandemic influenza preparedness. WHO is concerned with human health, FAO and OIE are concerned with animal health, while the World Bank is more concerned with the economic losses caused by the disease. Different main focus of these international organizations affected the content and article length of the documents regarding pandemic influenza preparedness. Fourth, the present bibliometric analysis focused on the official documents released by international organizations, and the documents released by WHO were treated as the gold standard. However, the choice of WHO text was also a kind of limitation, because pandemic influenza preparedness varied in different situations. For example, the US government has its own national implementation plan for pandemic influenza [31]. In terms of evaluation, a literature review by Arin Dutta provided a comprehensive description on determinants of a pandemic influenza outbreak, the effectiveness of different policies, cost and cost-effectiveness of policies and ethical dimensions of pandemic control policies [32]. Fifth, the phase during 2009 influenza pandemic was shorter than the other two phases before and after pandemic, which might cause the amount of those documents during the pandemic period was relatively smaller than those released in the other two periods, thus, the result of the word frequency analysis might be affected. Finally, the actual work was done 1–2 years earlier than the publication date of the included documents, which might mislead the assessment of the changes over time in the documents.

## Conclusions

Responsibilities of ministries of education, ministries of energy, ministries of agriculture and animal health, ministries of communication and the business sector in the pandemic influenza preparedness were described more comprehensively in the international organizations’ documents in 2017. Better understanding the variations of the guidelines delivered by international organizations would be useful for the member countries to strengthen their influenza control network.

## Additional file

Additional file 1: **Table S1.** Basic information of the included documents about pandemic influenza preparedness from international organization websites. (DOCX 30 kb)

## Abbreviations

ADPC: Asia-Pacific Regional Hub in collaboration with Asian Disaster Preparedness Center
AFP: Acute delayed paralysis
FAO: Food and Agriculture Organization of the United Nations
GPLN: Global polio laboratory network
GTZ: The German Technical Cooperation
ICAO: International Civil Aviation Organization
ILI: Influenza-like illness
ILO: International Labour Organization
IOM: International Organization for Migration
K. I. Asia: Kenan Institute Asia
OCHA: Office for the Coordination of Humanitarian Affairs
OIE: World Organization for Animal Health
TTX: Table-top exercise
UNDP: United Nations Development Programme
UNFPA: United Nations Population Fund
UNHCR: United Nations High Commissioner for Refugees
UNICEF: United Nations Children’s Fund
UNSIC: United Nations System Influenza Coordinator
UNWTO: World Tourism Organization
WFP: World Food Programme
WHO: World Health Organization
