# Supplementary material for: A review of documents prepared by international organizations about influenza pandemics, including the 2009 pandemic: a bibliometric analysis
Source: BMC Infect Dis. 2018 Aug 8;18:383. doi: 10.1186/s12879-018-3286-3 (PMC6083574; doi:10.1186/s12879-018-3286-3)
Supplement: Supplementary file 1 — Table S1. Basic information of the included documents about pandemic influenza preparedness from international organization websites. (DOCX 30 kb) [file 12879_2018_3286_MOESM1_ESM.docx]

**Table S1** Basic information of the included documents about pandemic influenza preparedness from international organization websites

| Number | Source | Author | Publishing Date | Title | Theme | Number of pages | Links |
| --- | --- | --- | --- | --- | --- | --- | --- |
| 1 | WHO | WHO | May 2017 | Pandemic Influenza Risk Management [1] | risk management | 62 | http://www.who.int/influenza/preparedness/pandemic/PIRM_update_052017.pdf?ua=1 |
| 2 | The World Bank | Olga Jonas  Lucas Warford | September 2014 | Global Program for Avian Influenza Control and Human Pandemic Preparedness and Response : Project Accomplishments [2] | project accomplishments | 112 | https://openknowledge.worldbank.org/bitstream/handle/10986/21541/940430WP0Box385430B0GPAI0Final00PUBLIC0.pdf?sequence=1&isAllowed=y |
| 3 | UNSIC | Olga B. Jonas (The World Bank) | October 2013 | Pandemic risk [3] | pandemic risk | 40 | http://un-influenza.org/sites/default/files/WDR14_bp_Pandemic_Risk_Jonas.pdf |
| 4 | UNSIC | WHO | October 2013 | Pandemic Influenza Risk Management: WHO Interim Guidance [4] | risk management | 62 | http://www.who.int/influenza/preparedness/pandemic/GIP_PandemicInfluenzaRiskManagementInterimGuidance_Jun2013.pdf?ua=1 |
| 5 | The World Bank | Monica Das Gupta  K.C.S Dalpatadu  C.K Shanmugarajah  H.M.S.S.D Herath | August 2013 | Multisectoral Preventive Health Services in Sri Lanka : Lessons for Developing Countries in Providing Public Goods in Health [5] | multisectoral preventive health services | 39 | https://openknowledge.worldbank.org/bitstream/handle/10986/21475/WPS6558.pdf?sequence=1&isAllowed=y |
| 6 | WHO | WHO | 2013 | Evolution of A Pandemic: A(H1N1) 2009, April 2009 – August 2010 – 2nd ed. [6] | evolution of a pandemic: A(H1N1) | 48 | http://apps.who.int/iris/bitstream/10665/78414/1/9789241503051_eng.pdf?ua=1 |
| 7 | UNSIC | Bile, Dr. Khalif Mohamud  Dunn, Mr Claude | April 2012 | The Central Fund for Influenza Action: Lessons Learned Exercise [7] | funds | 89 | http://un-influenza.org/sites/default/files/CFIALessonsLearned%20Report-May2012-1_0.pdf |
| 8 | UNSIC | UNSIC | October 2011 | United Nations Medical Directors Influenza Pandemic Guidelines [8] | medical directors | 46 | http://www.un-influenza.org/?q=content/pandemic-preparedness-guidelines |
| 9 | UNSIC | UNSIC | September 2011 | UN Medical Directors’ Guidelines on Establishment of A Fever Clinic [9] | medical directors’ guidelines | 4 | http://www.un-influenza.org/?q=content/un-medical-directors-guidelines-establishment-fever-clinic |
| 10 | WHO | WHO | July 2011 | Maintaining a Safe and Adequate Blood Supply during Pandemic Influenza [10] | blood supply | 8 | http://www.who.int/bloodsafety/transfusion_services/WHO_Guidelines_on_Pandemic_Influenza_and_Blood_Supply.pdf?ua=1 |
| 11 | UNSIC | Produced on behalf of FAO, ICAO, ILO, IOM, OCHA, OIE, UNDP, UNFPA, UNHCR, UNICEF, UNWTO, WFP and WHO by UNSIC | July 2011 | UN System and Partners Consolidated Action Plan for Animal and Human Influenza [11] | consolidated action plan | 28 | http://www.un-influenza.org/sites/default/files/UNCAPAHI-June2011.pdf |
| 12 | UNSIC | UNSIC | June 2011 | [Avian and Pandemic Influenza-related Programmes and Projects of the Inter-Governmental Entities in Asia and the Pacific](http://www.un-influenza.org/sites/default/files/Regional%20Inter-Goverenmental%20Entities%20API%20Initiatives%20(UNSIC).pdf) [12] | programmes and projects | 131 | http://www.un-influenza.org/sites/default/files/Regional%20Inter-Goverenmental%20Entities%20API%20Initiatives%20(UNSIC).pdf |
| 13 | UNSIC | UNSIC | July 2011 | UN system coordination for avian and pandemic influenza: Lessons on effective country coordination [13] | country coordination | 64 | http://www.un-influenza.org/sites/default/files/LessonsEffectiveCountryCoordination_UNSIC.pdf |
| 14 | WHO | WHO | May 2011 | Pandemic influenza preparedness Framework for the sharing of influenza viruses and access to vaccines and other benefits [14] | vaccines and other benefits | 68 | http://apps.who.int/iris/bitstream/10665/44796/1/9789241503082_eng.pdf |
| 15 | WHO | WHO | January 2011 | Comparative analysis of national pandemic influenza preparedness plans [15] | national pandemic influenza preparedness plans | 64 | http://www.who.int/influenza/resources/documents/comparative_analysis_php_2011_en.pdf?ua=1 |
| 16 | WHO | WHO | 2011 | Public health measures during the influenza A(H1N1)2009 pandemic [16] | public health measures | 44 | http://apps.who.int/iris/bitstream/10665/70747/1/WHO_HSE_GIP_ITP_2011.3_eng.pdf |
| 17 | UNSIC | WHO | 2011 | Assessment and treatment in the community during an influenza outbreak [17] | assessment and treatment | 2 | http://www.who.int/influenza/resources/documents/CCM_JobAid_web.pdf?ua=1 |
| 18 | The World Bank | United Nations, The World Bank | July 2010 | Animal and Pandemic Influenza : A Framework for Sustaining Momentum [18] | a framework for sustaining momentum | 180 | https://openknowledge.worldbank.org/bitstream/handle/10986/18202/879300PUB0Box30Progress0Report02010.pdf?sequence=1&isAllowed=y |
| 19 | UNSIC | WHO, UN Office for the Coordination of Humanitarian Affairs, UNSIC | 30 June 2010 | Urgent Support for Developing Countries' Responses to the H1N1 Influenza Pandemic [19] | urgent support(resources and activities) | 21 | http://www.un-influenza.org/sites/default/files/June2010UNIPReportFINAL.pdf |
| 20 | UNSIC | WHO, UN Office for the Coordination of Humanitarian Affairs, UNSIC | 31 March 2010 | Urgent Support for Developing Countries' Responses to the H1N1 Influenza Pandemic [20] | urgent support(resources and activities) | 38 | http://www.un-influenza.org/sites/default/files/FinalUNIPReportMarch2010.pdf |
| 21 | WHO | WHO | July 2009 | Whole-Of-Society Pandemic Readiness [21] | whole-of-society pandemic readiness | 18 | http://www.who.int/influenza/preparedness/pandemic/2009-0808_wos_pandemic_readiness_final.pdf?ua=1 |
| 22 | WHO | WHO | 2009 | Proposals to finalize remaining elements of the “Pandemic Influenza Preparedness Framework for sharing influenza viruses and access to vaccines and other benefits” [22] | proposals on vaccines and other benefits | 8 | http://www.who.int/influenza/resources/documents/hsegippip2009.1en.pdf?ua=1 |
| 23 | WHO | WHO | 2009 | Pandemic Influenza Preparedness and Response [23] | influenza preparedness and response | 64 | http://apps.who.int/iris/bitstream/10665/44123/1/9789241547680_eng.pdf?ua=1 |
| 24 | The World Bank | Arin Dutta | February 2008 | The Effectiveness of Policies to Control A Human Influenza Pandemic : A Literature Review [24] | effectiveness of policies | 57 | https://openknowledge.worldbank.org/bitstream/handle/10986/6397/wps4524.pdf?sequence=1&isAllowed=y |
| 25 | UNSIC | UNSIC, ADPC, K.I.Asia | 2008 | Simulation exercises on influenza pandemic responses in the Asia-Pacific region [25] | simulation exercises | 100 | http://un-influenza.org/sites/default/files/unsic_pandemic_complete.pdf |
| 26 | UNSIC | United Nations Office for the Coordination of Humanitarian Affairs | December 2007 | 39 Steps Governments Should Take to Prepare for A Pandemic [26] | prepare for a pandemic | 4 | http://www.un-influenza.org/sites/default/files/39-steps.pdf |
| 27 | UNSIC | Produced on behalf of FAO, ICAO, ILO, IOM, OCHA, OIE, UNDP, UNFPA, UNHCR, UNICEF, UNWTO, WFP and WHO by UNSIC | 17 September 2007 | Review of the Consolidated Action Plan for Contributions of the UN System and Partners (UNCAPAHI) [27] | review | 47 | http://www.un-influenza.org/sites/default/files/UNCAPAHIREVIEW2007NOV.pdf |
| 28 | UNSIC | UNSIC, GTZ, WHO | February 2007 | Coordination of Avian and Human Influenza Activities [28] | coordination of avian and human influenza activities | 45 | http://www.un-influenza.org/sites/default/files/STUDY-ON-COORDINATION-OF-AVIAN-AND-HUMAN-INFLUENZA.pdf |
| 29 | WHO | WHO | 2007 | WHO activities in avian influenza and pandemic influenza preparedness [29] | vavian influenza and pandemic influenza preparedness | 37 | http://www.un-influenza.org/sites/default/files/review_nov06_dec07.pdf |
| 30 | UNSIC | Produced on behalf of FAO, ICAO, ILO, IOM, OCHA, OIE, UNDP, UNHCR, UNICEF, UNWTO, WFP and WHO by UNSIC | 24 November 2006 | Consolidated Action Plan For Contributions of the UN System and Partners Revised Activities and Financial Requirements Up to December 2007 [30] | consolidated action plan | 48 | http://www.un-influenza.org/sites/default/files/review_july_dec06.pdf |
| 31 | UNSIC | Produced on behalf of FAO, OCHA, UNDP, UNHCR, UNICEF, WFP and WHO by UNSIC | 2 July 2006 | Avian and Human Pandemic Influenza: Consolidated Action Plan for UN System Contributions [31] | consolidated action plan | 48 | http://www.who.int/influenza/resources/documents/WHO_CDS_EPR_GIP_2006_6.pdf?ua=1 |
| 32 | WHO | WHO | 2006 | Global pandemic influenza action plan to increase vaccine supply [32] | vaccine supply | 24 | http://apps.who.int/iris/bitstream/10665/69388/1/WHO_IVB_06.13_eng.pdf |
| 33 | WHO | WHO | 2005 | WHO checklist for influenza pandemic preparedness planning [33] | preparedness planning | 39 | http://apps.who.int/iris/bitstream/10665/68980/1/WHO_CDS_CSR_GIP_2005.4.pdf |
| 34 | WHO | WHO | November 2004 | Vaccines for pandemic influenza [34] | vaccines | 21 |  |
| 35 | WHO | WHO | June 2004 | Informal consultation on influenza pandemic preparedness in countries with limited resources. Kuala Lumpur, Malaysia, 23-25 June 2004 [35] | influenza pandemic preparedness | 24 | http://www.who.int/influenza/resources/documents/CDS_CSR_GIP_2004_1.pdf |
| 36 | WHO | WHO | 2004 | WHO guidelines on the use of vaccines and antivirals during influenza pandemics [36] | vaccines and antivirals | 51 | http://www.who.int/influenza/resources/documents/11_29_01_A.pdf |
| 37 | WHO | WHO | unknown | Considerations on exercises to validate pandemic preparedness plans [37] | exercises | 1 | http://www.who.int/influenza/resources/documents/ExerciseConsiderations.pdf |
| 38 | UNSIC | UN Office for the Coordination of Humanitarian Affairs | unknown | Recommendations to Governments on the prioritisation of “other essential services personnel’’ for H1N1 vaccine [38] | H1N1 vaccine | 5 | http://www.un-influenza.org/sites/default/files/otherpersonnelH1N1vaccine.EN.pdf |

**References**

1. World Health Organization. Pandemic Influenza Risk Management. Geneva: World Health Organization; 2017. http://www.who.int/influenza/preparedness/pandemic/PIRM_update_052017.pdf?ua=1. Accessed 3 July 2017

2. Jonas O, Warford L. Global Program for Avian Influenza Control and Human Pandemic Preparedness and Response: Project Accomplishments. Washington, DC: The International Bank for Reconstruction and Development / The World Bank; 2014. https://openknowledge.worldbank.org/bitstream/handle/10986/21541/940430WP0Box385430B0GPAI0Final00PUBLIC0.pdf?sequence=1&isAllowed=y. Accessed 3, July 2017

3. Jonas OB. Pandemic risk. Washington, DC: the World Bank; 2013. http://un-influenza.org/sites/default/files/WDR14_bp_Pandemic_Risk_Jonas.pdf. Accessed 3 July 2017

4. World Health Organization. Pandemic Influenza Risk Management. Geneva: World Health Organization; 2013. http://www.who.int/influenza/preparedness/pandemic/GIP_PandemicInfluenzaRiskManagementInterimGuidance_Jun2013.pdf?ua=1. Accessed 3 July 2017

5. Gupta MD, Dalpatadu KCS, Shanmugarajah CK, Herath HMSS. Multisectoral Preventive Health Services in Sri Lanka : Lessons for Developing Countries in Providing Public Goods in Health. Washington, DC: the World Bank; 2013. https://openknowledge.worldbank.org/bitstream/handle/10986/21475/WPS6558.pdf?sequence=1&isAllowed=y. Accessed 3 July 2017

6. World Health Organization. Evolution of a pandemic: A(H1N1) 2009, April 2009 – August 2010 – 2nd ed. Geneva: World Health Organization; 2013. http://apps.who.int/iris/bitstream/10665/78414/1/9789241503051_eng.pdf?ua=1. Accessed 3 July 2017

7. Bile DKM, Dunn MC. The Central Fund for Influenza Action: Lessons Learned Exercise. New York: UN System Influenza Coordination Office (UNSIC); 2012. http://un-influenza.org/sites/default/files/CFIALessonsLearned%20Report-May2012-1_0.pdf. Accessed 3 July 2017

8. United Nations System Influenza Coordinator. United Nations Medical Directors Influenza Pandemic Guidelines. New York: UN System Influenza Coordination Office (UNSIC); 2011. http://www.un-influenza.org/?q=content/pandemic-preparedness-guidelines. Accessed 3 July 2017

9. United Nations System Influenza Coordinator. UN Medical Directors’ Guidelines on Establishment of A Fever Clinic. New York: UN System Influenza Coordination Office (UNSIC); 2011. http://www.un-influenza.org/?q=content/un-medical-directors-guidelines-establishment-fever-clinic. Accessed 3 July 2017

10. World Health Organization. Maintaining a Safe and Adequate Blood Supply during Pandemic Influenza. Geneva: World Health Organization; 2011. http://www.who.int/bloodsafety/transfusion_services/WHO_Guidelines_on_Pandemic_Influenza_and_Blood_Supply.pdf?ua=1. Accessed 3 July 2017

11. United Nations System Influenza Coordinator. UN System and Partners Consolidated Action Plan for Animal and Human Influenza. New York: UN System Influenza Coordination Office (UNSIC); 2011. http://www.un-influenza.org/sites/default/files/UNCAPAHI-June2011.pdf. Accessed 3 July 2017

12. United Nations System Influenza Coordinator. Avian and Pandemic Influenza-related Programmes and Projects of the Inter-Governmental Entities in Asia and the Pacific. New York: UN System Influenza Coordination Office (UNSIC); 2011. http://www.un-influenza.org/sites/default/files/Regional%20Inter-Goverenmental%20Entities%20API%20Initiatives%20(UNSIC).pdf. Accessed 3 July 2017

13. United Nations System Influenza Coordinator. UN system coordination for avian and pandemic influenza: Lessons on effective country coordination. New York: UN System Influenza Coordination Office (UNSIC); 2011. http://www.un-influenza.org/sites/default/files/LessonsEffectiveCountryCoordination_UNSIC.pdf. Accessed 3 July 2017

14. World Health Organization. Pandemic influenza preparedness Framework for the sharing of influenza viruses and access to vaccines and other benefits. Geneva: World Health Organization; 2011. http://apps.who.int/iris/bitstream/10665/44796/1/9789241503082_eng.pdf. Accessed 3 July 2017

15. World Health Organization. Comparative analysis of national pandemic influenza preparedness plans. Geneva: World Health Organization; 2011. http://www.who.int/influenza/resources/documents/comparative_analysis_php_2011_en.pdf?ua=1. Accessed 3 July 2017

16. World Health Organization. Public health measures during the influenza A(H1N1)2009 pandemic. Geneva: World Health Organization; 2011. http://apps.who.int/iris/bitstream/10665/70747/1/WHO_HSE_GIP_ITP_2011.3_eng.pdf. Accessed 3 July 2017

17. World Health Organization. Assessment and treatment in the community during an influenza outbreak. Geneva: World Health Organization; 2011. http://www.who.int/influenza/resources/documents/CCM_JobAid_web.pdf?ua=1. Accessed 3 July 2017

18. Nations U, Bank TW. Animal and Pandemic Influenza: A Framework for Sustaining Momentum; 2010. https://openknowledge.worldbank.org/bitstream/handle/10986/18202/879300PUB0Box30Progress0Report02010.pdf?sequence=1&isAllowed=y. Accessed 3 July 2017

19. World Health Organization, United Nations Office for the Coordination of Humanitarian Affairs, United Nations System Influenza Coordinator. Urgent Support for Developing Countries' Responses to the H1N1 Influenza Pandemic; 2010. http://www.un-influenza.org/sites/default/files/June2010UNIPReportFINAL.pdf. Accessed 3 July 2017

20. World Health Organization, United Nations Office for the Coordination of Humanitarian Affairs, United Nations System Influenza Coordinator. Urgent Support for Developing Countries' Responses to the H1N1 Influenza Pandemic; 2010. http://www.un-influenza.org/sites/default/files/FinalUNIPReportMarch2010.pdf. Accessed 3 July 2017

21. World Health Organization. Whole-Of-Society Pandemic Readiness. Geneva: World Health Organization; 2009. http://www.who.int/influenza/preparedness/pandemic/2009-0808_wos_pandemic_readiness_final.pdf?ua=1. Accessed 3 July 2017

22. World Health Organization. Proposals to finalize remaining elements of the “Pandemic Influenza Preparedness Framework for sharing influenza viruses and access to vaccines and other benefits”. Geneva: World Health Organization; 2009. http://www.who.int/influenza/resources/documents/hsegippip2009.1en.pdf?ua=1. Accessed 3 July 2017

23. World Health Organization. Pandemic Influenza Preparedness and Response. Geneva: World Health Organization; 2009. http://apps.who.int/iris/bitstream/10665/44123/1/9789241547680_eng.pdf?ua=1. Accessed 3 July 2017

24. Dutta A. The Effectiveness of Policies to Control a Human Influenza Pandemic : A Literature Review. Washington, DC: the World Bank; 2008. https://openknowledge.worldbank.org/bitstream/handle/10986/6397/wps4524.pdf?sequence=1&isAllowed=y. Accessed 3 July 2017

25. United Nations System Influenza Coordinator, Asia-Pacific Regional Hub in collaboration with Asian Disaster Preparedness Center, Kenan Institute Asia. Simulation exercises on influenza pandemic responses in the Asia-Pacific region. New York: UN System Influenza Coordination Office (UNSIC); 2008. http://un-influenza.org/sites/default/files/unsic_pandemic_complete.pdf

26. United Nations Office for the Coordination of Humanitarian Affairs. 39 Steps Governments Should Take to Prepare for A Pandemic. New York: the United Nations; 2007. http://www.un-influenza.org/sites/default/files/39-steps.pdf. Accessed 3 July 2017

27. United Nations System Influenza Coordinator. Review of the Consolidated Action Plan for Contributions of the Un System and Partners (UNCAPAHI). New York: UN System Influenza Coordination Office (UNSIC); 2007. http://www.un-influenza.org/sites/default/files/UNCAPAHIREVIEW2007NOV.pdf. Accessed 3 July 2017

28. United Nations System Influenza Coordinator, The German Technical Cooperation, World Health Organization. Coordination of Avian and Human Influenza Activities; 2007. http://www.un-influenza.org/sites/default/files/STUDY-ON-COORDINATION-OF-AVIAN-AND-HUMAN-INFLUENZA.pdf. Accessed 3 July 2017

29. World Health Organization. WHO activities in avian influenza and pandemic influenza preparedness. Geneva: World Health Organization; 2007. http://www.un-influenza.org/sites/default/files/review_nov06_dec07.pdf. Accessed 3 July 2017

30. United Nations System Influenza Coordinator. Consolidated Action Plan for Contributions of the UN System and Partners Revised Activities and Financial Requirements Up to December 2007. New York: UN System Influenza Coordination Office (UNSIC); 2006. http://www.un-influenza.org/sites/default/files/review_july_dec06.pdf. Accessed 3 July 2017

31. United Nations System Influenza Coordinator. Avian and Human Pandemic Influenza: Consolidated Action Plan for UN System Contributions. New York: UN System Influenza Coordination Office (UNSIC); 2006. http://www.who.int/influenza/resources/documents/WHO_CDS_EPR_GIP_2006_6.pdf?ua=1. Accessed 3 July 2017

32. World Health Organization. Global pandemic influenza action plan to increase vaccine supply. Geneva: World Health Organization; 2006. http://apps.who.int/iris/bitstream/10665/69388/1/WHO_IVB_06.13_eng.pdf. Accessed 3 July 2017

33. World Health Organization. WHO checklist for influenza pandemic preparedness planning. Geneva: World Health Organization; 2005. http://apps.who.int/iris/bitstream/10665/68980/1/WHO_CDS_CSR_GIP_2005.4.pdf

34. World Health Organization. Vaccines for pandemic influenza. Geneva: World Health Organization; 2004. Accessed 3 July 2017

35. World Health Organization. Informal consultation on influenza pandemic preparedness in countries with limited resources. Kuala Lumpur, Malaysia, 23-25 June 2004. Geneva: World Health Organization; 2004. http://www.who.int/influenza/resources/documents/CDS_CSR_GIP_2004_1.pdf. Accessed 3 July 2017

36. World Health Organization. WHO guidelines on the use of vaccines and antivirals during influenza pandemics. Geneva: World Health Organization; 2004. http://www.who.int/influenza/resources/documents/11_29_01_A.pdf. Accessed 3 July 2017

37. World Health Organization. Considerations on exercises to validate pandemic preparedness plans. Geneva: World Health Organization. http://www.who.int/influenza/resources/documents/ExerciseConsiderations.pdf. Accessed 3 July 2017

38. United Nations Office for the Coordination of Humanitarian Affairs. Recommendations to Governments on the prioritisation of "other essential services personnel" for H1N1 vaccine. New York: the United Nations. http://www.un-influenza.org/sites/default/files/otherpersonnelH1N1vaccine.EN.pdf. Accessed 3 July 2017
